# AML classification in the year 2023: How to avoid a Babylonian confusion of languages

**DOI:** 10.1038/s41375-023-01909-w

**Published:** 2023-04-29

**Authors:** Sandra Huber, Constance Baer, Stephan Hutter, Frank Dicker, Manja Meggendorfer, Christian Pohlkamp, Wolfgang Kern, Torsten Haferlach, Claudia Haferlach, Gregor Hoermann

**Affiliations:** MLL Munich Leukemia Laboratory, Max-Lebsche-Platz 31, 81377 Munich, Germany

**Keywords:** Acute myeloid leukaemia, Acute myeloid leukaemia

## Abstract

In parallel to the 5th edition of the World Health Organization Classification of Haematolymphoid Tumours (WHO 2022), an alternative International Consensus Classification (ICC) has been proposed. To evaluate the impact of the new classifications on AML diagnoses and ELN-based risk classification, we analyzed 717 MDS and 734 AML non-therapy-related patients diagnosed according to the revised 4th WHO edition (WHO 2017) by whole genome and transcriptome sequencing. In both new classifications, the purely morphologically defined AML entities decreased from 13% to 5%. Myelodysplasia-related (MR) AML increased from 22% to 28% (WHO 2022) and 26% (ICC). Other genetically-defined AML remained the largest group, and the abandoned AML-*RUNX1* was mainly reclassified as AML-MR (WHO 2022: 77%; ICC: 96%). Different inclusion criteria of AML-*CEBPA* and AML-MR (*i.a*. exclusion of *TP53* mutated cases according to ICC) were associated with differences in overall survival. In conclusion, both classifications focus on more genetics-based definitions with similar basic concepts and a large degree of agreement. The remaining non-comparability (e.g., *TP53* mutated AML) needs additional studies to definitely answer open questions on disease categorization in an unbiased way.

## Introduction

Myeloid malignancies have so far been classified according to the revised 4th edition of World Health Organization Classification of Haematolymphoid Tumours, published in 2017 (WHO 2017, [1]) - the state of the art for the diagnosis of leukemia and lymphoma. However, substantive advances in the field of genetics led to dynamic changes regarding the definition of specific sub-entities. Thus, the up-coming 5th edition of WHO Classification (WHO 2022, [2]), with a beta version already available online, highlights the genetic background for defining diseases. AML is now mainly divided into AML with defining genetic abnormalities (DGA) and AML defined by differentiation. Major changes compared to the previous edition are the broadening of *KMT2A* and *MECOM* rearrangements (-r), inclusion of *NUP98* and other DGAs and abandoning the previous category of AML with *RUNX1* mutation. The definition of AML with myelodysplasia-related changes (AML-MRC) has been modified – mainly abandoning the morphologic criteria of dysplasia and newly incorporating molecular genetics (defining somatic mutations: *ASXL1, BCOR, EZH2, SF3B1, SRSF2, STAG2, U2AF1, ZRSR2* based on previous studies [3, 4]). Subsequently, AML myelodysplasia-related (AML-MR) is now included into genetically defined AML. The biggest difference is the removal of the blast cutoff for all genetically defined AML cases but AML with *BCR*::*ABL1*, AML with *CEBPA* mutation, and AML-MR. In contrast, the 20% blast cutoff is still in place for AML defined by differentiation discriminating it from MDS.

In parallel to the WHO, an international expert panel proposed an independent classification named ICC for International Consensus Classification [5]. In contrast to the WHO 2022, ICC sets the blast cutoff for AML-DGA to 10%, assigning cases with 10–19% blasts without DGA to a new category MDS/AML. Another major difference between WHO 2022 and ICC is the introduction of the new entity AML with mutated *TP53*, which has been included in the ICC, but not in the WHO. In addition, with respect to *CEBPA* mutated AML, ICC only includes in-frame bZIP *CEBPA* mutations, while WHO 2022 accepts biallelic *CEBPA* mutations (as in WHO 2017) as well as single mutations of any kind that are located in the bZIP region of the gene. ICC also includes *KMT2A-* and *MECOM*-r with other partner genes than *MLLT3* or *GATA2* but specifically defines the partner genes. With respect to AML-MR, ICC separates this entity into AML with MR gene mutations (here including *RUNX1*) and with MR cytogenetic abnormalities, with mutations having the higher hierarchy. In contrast to WHO 2022, ICC does not consider a documented MDS or MDS/MPN history for diagnosing AML-MR but uses it as a diagnostic qualifier.

These changes described by ICC regarding AML classification are also mainly reflected within ELN 2022 recommendations for the diagnosis and management of AML in adults, guidelines for risk prediction in AML [6]. Here, depending on the presence of certain genetic abnormalities, AML patients are categorized into favorable, intermediate and adverse risk groups. Compared to the previous ELN 2017 edition [7] changes affected all three risk groups. Other *MECOM*-r and MR-defining gene mutations are added to the adverse risk-defining genetic abnormalities in ELN 2022. The *FLT3*-ITD allelic ratio used in ELN 2017 has been abolished in ELN 2022 risk classification mainly affecting *NPM1* mutated patients. Within the favorable *CEBPA* mutated risk group, ELN 2017 considers biallelic *CEBPA* mutations, but ELN 2022 only includes bZIP in-frame mutated *CEBPA* in line with ICC.

The aim of our study was to evaluate the impact of the new WHO classification on AML patients and to quantify differences in disease categorization compared to the previous WHO classification as well as the ICC.

## Material and methods

### Patient cohort

For this analysis we selected 1,451 non-therapy-related MDS or AML samples (MDS: *n* = 717; AML: *n* = 734) with material available to perform whole genome and transcriptome sequencing sent to the MLL Munich Leukemia Laboratory between 09/2005 and 01/2020. Demographic, pathologic, and clinical characteristics of the cohort are described in Table 1 and Supplementary Table S1. Clinical data was provided by the treating physicians upon request and was updated at least annually. All samples were subjected to whole genome and transcriptome sequencing (WGS: median coverage 100x; WTS: median yield 50 million reads; Supplementary Methods) and classified according to WHO 2017. For abbreviations of entities, see Supplementary Table S1. All patients gave their written informed consent for genetic analyses and to the use of laboratory results and clinical data for research purposes according to the Declaration of Helsinki. The study was further approved by the laboratory´s institutional review board.

**Table 1:** Patients characteristics.

	AML	MDS
Patients (*n*)	734	717
**Demographics**		
Female (*n*; %)	327 (44.6%)	303 (42.3%)
Age (yrs; median ± IQR)	68.4 (54.3–75.8)	73.2 (66.5–78.1)
Therapy-related (*n*)	0	0
Post-MDS or MDS/MPN (*n*; %)	45 (6.1%)	.
**Pathologic characteristics**		
Cytomorphology - availability (*n*; %)	732 (99.7%)	717 (100%)
BM blast count (%; median ± IQR)	66.0 (38.5-82.0)	4.0 (2.0-8.0)
Cytogenetics - availability	734 (100%)	716 (99.9%)
Aberrant karyotype (*n*; %)	465 (63.4%)	312 (43.6%)
Normal karyotype (*n*; %)	269 (36.6%)	404 (56.4%)
Molecular genetics - availability (*n*; %)	734 (100%)	717 (100%)
Mutation present (*n*; %)	728 (99.2%)	627 (87.5%)
*NPM1* mutation (*n*; %)	167 (22.8%)	6 (0.8%)
*FLT3*-ITD (*n*; %)	136 (18.5%)	5 (0.7%)
*CEBPA* mutation (*n*; %)	75 (10.2%)	16 (2.2%)
*RUNX1* mutation (*n*; %)	95 (12.9%)	66 (9.2%)
*TP53* mutation (*n*; %)	62 (8.4%)	80 (11.2%)
MR mutation (*n*; %)	255 (34.7%)	459 (64.0%)
**Clinical data**		
Survival data - availability (*n*; %)	687 (93.6%)	710 (99.1%)
Deceased patients (*n*; %)	452 (65.8%)	489 (68.9%)
Median follow-up (yrs)	8.3	10.4
Treatment data - availability (*n*; %)	642 (87.5%)	668 (93.2%)
Intensive chemotherapy (*n*; %)	452 (70.4%)	50 (7.5%)
Allogeneic HSCT (*n*; %)	157 (24.5%)	35 (5.2%)
Not intensive chemotherapy (*n*; %)	110 (17.1%)	185 (27.7%)
Unspecified (*n*; %)	12 (1.9%)	2 (0.3%)
Supportive treatment (*n*; %)	54 (8.4%)	242 (36.2%)
None (*n*; %)	14 (2.2%)	189 (28.5%)
Response data - availability (*n*; %)	630 (85.8%)	562 (78.4%)
CR reached (*n*; %)	354 (56.2%)	44 (7.8%)
Progress to AML (*n*; %)	.	110 (15.3%)

*IQR* Interquartile range, *BM* Bone marrow, *MR* Myelodysplasia-related (*ASXL1*, *BCOR*, *EZH2*, *SF3B1*, *SRSF2*, *STAG2*, *U2AF1*, *ZRSR2*), *HSCT* Hematopoietic stem cell transplantation, *CR* Complete remission.

### Statistical analysis

For statistical analyses SPSS version 19.0 (IBM Corporation, Armonk, NY) was used. Analyses for overall survival (OS) were performed according to Kaplan-Meier and compared using two-sided log rank tests. The OS was calculated as time from diagnosis to death or last follow-up. To assess the correlation between categories with real outcomes we used the Harrell’s concordance index (c-index [8]). All results were considered significant at *p* < 0.05.

## Results

### AML diagnoses – from WHO 2017 to WHO 2022

**Fig. 1:**
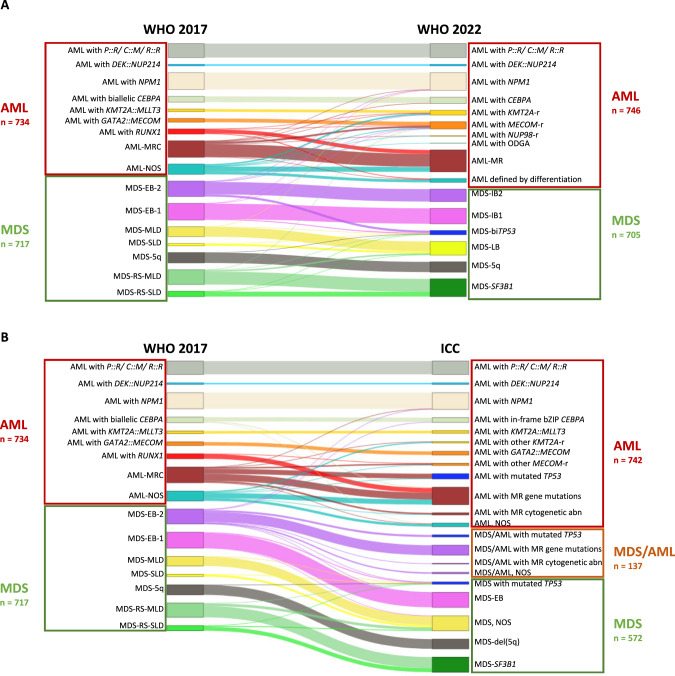
Changes in specific MDS and AML diagnoses compared to WHO 2017. Changes in diagnoses according to WHO 2022 (**A**) or ICC (**B**). *P::R* = *PML::RARA*; *C::M* = *CBFB::MYH11*; *R::R* = *RUNX1::RUNX1T1*; MR(C) Myelodysplasia-related (changes), NOS Not otherwise specified, EB Excess blasts, SLD Single lineage dysplasia, MLD Multilineage dysplasia; 5q/del(5q) Isolated 5q deletion, RS Ring sideroblasts; -r rearrangement; ODGA Other defined genetic alterations, IB Increased blasts, bi*TP53* biallelic *TP53* inactivation, LB Low blasts, abn Abnormalities.

According to WHO 2022, 746 patients were diagnosed as AML and 705 patients as MDS (Fig. 1A, Supplementary Table S2). Within the AML cases, genetically defined AML (excluding here AML-MRC/AML-MR for comparability with WHO 2017) was the largest subgroup with 65% (477/734) and 67% (502/746) of AML cases in both WHO classification systems, respectively. However, the composition of the genetically defined AML changed. Notably, APL, core binding factor AML, and AML with *DEK::NUP214* fusion did not change at all. Major additional contributors were AML with *KMT2A*-r (*n* = 45) now including 19 cases (42%) with a different partner gene but *MLLT3* (Supplementary Fig. S1) and AML with *MECOM*-r (*n* = 69) now including 33 cases (48%) not comprising *GATA2* (Supplementary Fig. S2). In addition, the newly recognized entities included 6 cases, 5 belonging to AML with *NUP98* rearrangement (partner genes *KDM5A*: *n* = 2, *NSD1*: *n* = 2, *STIM1*: *n* = 1) and one case to AML with another defined genetic aberration (*KAT6A*::*CREBBP)*. In contrast, the now abandoned entity AML with mutated *RUNX1* was mainly reclassified as AML-MR (37/48; 77%) based on a large overlap with *ASXL1* and splicing factor mutations (Supplementary Fig. S3). Only 7 cases of this former category were not otherwise genetically defined and moved to AML defined by differentiation. In addition, 10 further cases were classified as AML with mutated *NPM1* (according to WHO 2017: AML-MRC: *n* = 4; MDS-EB-2: *n* = 4; MDS-EB-1: *n* = 1; MDS-MLD: *n* = 1) and 5 cases as AML with mutated *CEBPA* (former AML-MRC due to the superior hierarchical status of a previously documented MDS or MDS/MPN history or showing MR cytogenetic abnormalities in the WHO 2017).

Myelodysplasia-related AML substantially increased from 22% (158/734) AML-MRC to 28% (208/746) AML-MR in WHO 2022 (Supplementary Fig. S4A; 122/208 former AML-MRC, 49/208 former AML-NOS, 37/208 former AML-*RUNX1*). The largest contributor to this increase were mutations in the defining genes solely leading to this classification in 44% (92/208) of cases (Supplementary Fig. S4B). Notably, cyto- and molecular genetics without medical history were sufficient for AML-MR classification in all patients. In addition, 23% (36/158) former AML-MRC patients were not re-classified as AML-MR: 33 harbored defining genetic abnormalities (55% with *MECOM*-r: *n* = 18) and three were defined by differentiation as the morphologic dysplasia had been the only MRC-defining criterion according to WHO 2017 (Fig. 1A). Complementary to these findings, the morphologically defined group was reduced from 13% (99/734) AML-NOS to 5% (36/746) AML with differentiation (Supplementary Fig. S5). When stratified into morphologically defined subgroups, the decrease was particularly striking for acute monoblastic and monocytic leukemia.

Overall, reclassification from MDS according to WHO 2017 to AML according to WHO 2022 was a rare event affecting < 1% of cases of the total cohort (2% of the MDS cohort; Supplementary Table S3). In total, 12 former MDS samples, 8 of them EB-2, were upstaged to AML based on DGA (*MECOM*-r: *n* = 5; *KMT2A*-r: *n* = 1; *NPM1*: *n* = 6).

### AML diagnoses – from WHO 2017 to ICC

When following ICC, the cohort comprised 742 AML, 572 MDS, and 137 MDS/AML cases (Fig. 1B; Supplementary Table S4). This new category of MDS/AML overlap is the largest change from WHO 2017 to ICC affecting 9% of all patients. Focusing on AML cases, 69% (515/742) were genetically defined AML (excluding here again AML-MR). The increase from 65% based on WHO 2017 to 69% following ICC was mainly mediated by introducing the new entity AML with mutated *TP53* comprising 52 cases of which the majority was former AML-MRC (48/52; 92%). Within *TP53* mutated AML we observed a high fraction of cases with complex karyotype and biallelic *TP53* mutations but only a weak overlap with MR mutations (Supplementary Table S5). In addition, AML with other *KMT2A*-r but *MLLT3* and other *MECOM*-r but *GATA2* were introduced including 14 and 21 cases, respectively (Supplementary Fig. S1 and S2).

Regarding AML-MR, in our cohort, 174 cases harbored MR gene mutations while 19 showed certain MR cytogenetics (in total 193/746, 26% of AML cases). AML with MR gene mutations mainly composed former AML-MRC (*n* = 69), AML-NOS (*n* = 52) and AML-*RUNX1* (*n* = 46). As in ICC – in contrast to WHO 2022 – *RUNX1* mutations were qualifying mutations for AML-MR, 46 of the previous 48 (96%) AML-*RUNX1* cases were classified as AML-MR while two had other *MECOM*-r. In line, AML-NOS was reduced from 99 to 34 cases (5%) not further characterized with respect to morphology (Supplementary Fig. S5). In addition, according to ICC criteria, 8 former MDS-EB-2 cases were upstaged to AML due to *NPM1* or in-frame bZIP *CEBPA* mutations (Supplementary Table S3).

### Survival analysis of AML entities according to different classification systems

**Fig. 2:**
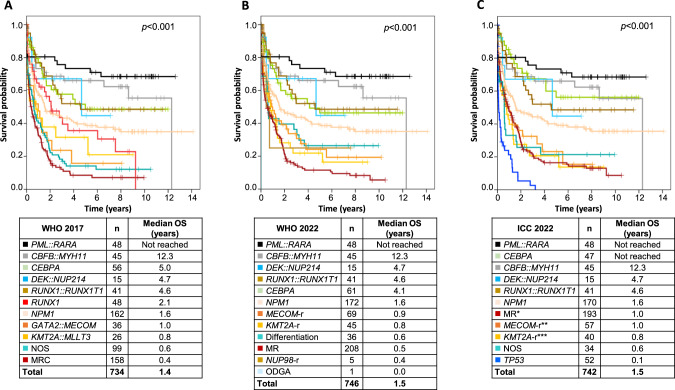
Overall survival (OS) of AML patients according to different classifications. **A** OS of AML patients according to WHO 2017 (*n* = 734). **B** OS of AML patients according to WHO 2022 (*n* = 746). **C** OS of AML patients according to ICC (*n* = 742). NOS: not otherwise specified; MR(C): myelodysplasia-related (changes); -r rearrangement, ODGA other defined genetic alterations, *comprises MR gene mutations and MR cytogenetic abnormalities; **comprises *GATA2::MECOM* and other *MECOM*-r; **comprises *KMT2A::MLLT3* and other *KMT2A*-r.

Kaplan-Meier analyses of overall survival (OS) revealed marked differences between AML entities classified according to WHO 2017, WHO 2022 and ICC. For all classifications prognostic significance was confirmed (Fig. 2; overall *p* < 0.001). Across the different classifications, major changes regarding OS were observed for AML-*CEBPA* and AML-MR both entities present in all classification systems but with different inclusion criteria. *CEBPA* mutated AML showed a median OS of 5.0 and 4.1 years based on WHO 2017 and WHO 2022, respectively, while it was not reached according to ICC. Notably, based on WHO 2017 this entity comprised cases with biallelic *CEBPA* mutations (*n* = 56), WHO 2022 additionally considered single *CEBPA* mutations located in bZIP region (*n* = 61; in our cohort all with biallelic mutation; 5 additional biallelic *CEBPA* mutated cases were former AML-MRC due to differences in entity hierarchy) and ICC only included in-frame bZIP mutations (*n* = 47). Concerning myelodysplasia-related AML, AML-MRC based on WHO 2017 had the shortest median OS with 0.4 years compared to AML-MR according to WHO 2022 with a median OS of 0.5 years and AML-MR according to ICC with a median OS of 1.0 years. The later was mediated by excluding *TP53* mutated cases which showed the worst OS (0.1 years) within all AML entities. Within *TP53* mutated cases we observed a trend towards effects of *TP53* mutation status (mono- vs. biallelic) on OS (Supplementary Fig. S6). When differentiating AML with MR cytogenetic abnormalities from AML with MR gene mutations according to ICC, no difference in OS was observed (median OS: 0.71 vs 0.98 years; *p* = 0.958; Supplementary Fig. S7).

### Heterogeneity of AML entities between new classification systems

**Fig. 3:**
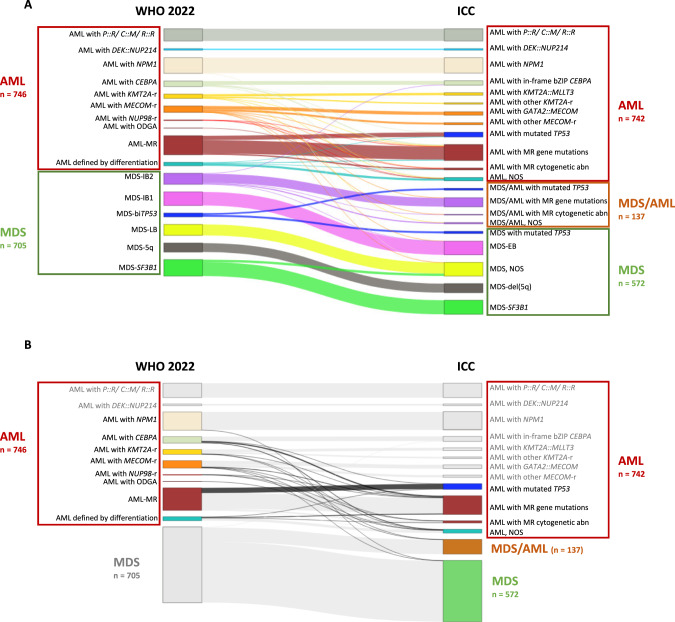
Changes in AML diagnoses between WHO 2022 and ICC. **A** Changes in specific MDS and AML diagnoses between WHO 2022 and ICC. **B** Major differences in AML diagnoses between WHO 2022 and ICC. *P::R* = *PML::RARA*; *C::M* = *CBFB::MYH11*; *R::R* = *RUNX1::RUNX1T1*; MR Myelodysplasia-related, NOS Not otherwise specified, EB Excess blasts, SLD Single lineage dysplasia, MLD Multilineage dysplasia, 5q/del(5q) Isolated 5q deletion, RS Ring sideroblasts, -r rearrangement, ODGA Other defined genetic alterations, IB Increased blasts, bi*TP53* Biallelic *TP53* inactivation, LB Low blasts, abn Abnormalities.

A direct comparison of the ICC with the WHO 2022 highlighted several differences (Fig. 3A). Overall, the classification of the disease subgroup differed in 14% (104/746) of AML cases (not taking the category of MDS/AML into account; Fig. 3B). While some of these changes arise from minor differences in subtype definitions, others reflect major differences in the two classification systems – including an additional biological subgroup in the ICC (AML with mutated *TP53*).

*KMT2A*- and *MECOM*-rearranged cases formed two entities in WHO 2022 but were split into four different entities following ICC separating other partner genes than *MLLT3* or *GATA2*. However, there was no significant difference in OS between *KMT2A::MLLT3* and other *KMT2A*-r cases (Supplementary Fig. S1). The same was true for the survival of *GATA2::MECOM* and other *MECOM*-r cases (Supplementary Fig. S2). Major differences also affected myelodysplasia-related AML (Supplementary Fig. S8). *TP53* mutations were detected in 23% (48/208) of AML-MR leading to a classification as AML-*TP53* according to ICC. Notably, AML-*TP53* showed shorter OS compared to AML-MR according to WHO 2022 (median OS: 0.1 vs 1.0 years, *p* < 0.001; Supplementary Fig. S9).

The different definitions for the upstaging from MDS to AML according to genetics and blast cutoffs led to 12 patients with differences in the main diagnosis – AML or MDS – between the two new classifications (Supplementary Table S3). Thus, the upstaging from MDS to AML was not consistent between WHO 2022 and ICC. Only 4 of total 16 cases were concordantly upstaged to AML in both new classifications.

The largest difference between WHO 2022 and ICC was mediated by the introduction of the new category MDS/AML including mainly MDS-IB2, but also MDS and AML cases (MDS-bi*TP53* and AML with *MECOM*-r; Supplementary Fig. S10). MDS/AML separated into those with *TP53* mutations (19/137; 14%), with MR gene mutations (99/137; 72%), with MR cytogenetic abnormalities (6/137; 4%) and MDS/AML, NOS (13/137; 10%).

### Risk stratification based on ELN 2017 and ELN 2022

**Fig. 4:**
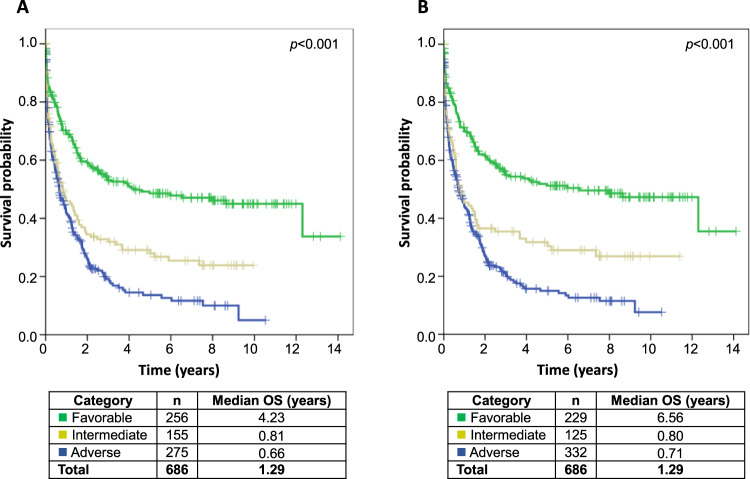
OS of AML patients according to ELN risk classifications. OS of AML patients based on WHO 2017 (*n* = 686; excluding AML with *PML::RARA*) according to ELN 2017 (**A**) or ELN 2022 (**B**) risk classifications. C-indices: 0.5967 for ELN 2017; 0.5961 for ELN 2022.

In line with AML disease classification according to ICC, also AML risk classification has been adjusted in ELN 2022 guidelines. In our cohort, favorable and intermediate groups shrunk from 256 to 229 and from 155 to 125 cases, respectively, while in contrast the adverse risk group increased from 275 to 332 cases (Supplementary Fig. S11, Supplementary Table S6). These changes within the criteria for the different risk groups did not only affect the group size, but were also to some degree reflected in the outcome data (Fig. 4). This impacted mainly the favorable risk category with a median OS of 6.6 years in ELN 2022 compared to 4.2 years in ELN 2017. The median OS of the intermediate groups was comparable (0.81 and 0.80 years), whereas of adverse groups slightly increased from ELN 2017 to ELN 2022 (0.66 and 0.71 years, respectively). However, c-indices (calculated to assess the correlation between predictions according to ELN 2017 and ELN 2022 with real outcomes) were similar between both ELN 2017 and ELN 2022 with 0.5967 and 0.5961, respectively.

## Discussion

In this comprehensive study of 1451 patients analyzed by WGS and WTS, we analyzed to which extent the new classification systems for myeloid malignancies impact the diagnosis of AML (or MDS). The general concept of a more genetics based definition of AML entities is consistent between the new classification systems substantially reducing purely morphologically defined cases. Despite this and many other concordant definitions and entity criteria between ICC and WHO 2022, there are several key differences that could influence patients’ diagnoses and risk stratification as well as therapeutic decisions and clinical trials in AML.

One point is the discrepant definition of *CEBPA* mutated AML. While WHO 2022 includes biallelic *CEBPA* mutations (independent of the gene region) and single mutations located in the bZIP region, ICC only accepts in-frame bZIP *CEBPA* mutations (independent of the allelic state). The inclusion of monoallelic bZIP mutations is supported by several recent studies [9–11]. The narrow definition of the ICC is based on data indicating that only in-frame bZIP mutations were associated with favorable clinical response in mono- and biallelic constellations [9]. Wakita et al. found similar results for bZIP mutations but did not specifically limit their analysis to the in-frame type [10]. Tarlock et al. showed no significant difference in event-free survival between AML patients with monoallelic bZIP and *CEBPA* double-mutant patients (defined as a second mutation in addition to a bZIP mutation) [11]. Indeed, patients following ICC criteria showed longer median OS in our cohort compared to patients falling into the WHO-based *CEBPA* mutated entities. Thus, regarding outcome of *CEBPA* mutated AML patients, the prerequisite of an (in-frame) bZIP mutation seems reasonable.

In contrast, discriminating *KMT2A*-r and *MECOM*-r cases from *MLLT3*- and *GATA2*- rearranged cases and accepting only specific partner genes according to ICC did not show differences regarding OS neither within rearranged cases nor compared to WHO 2022. Our findings regarding *MECOM*-r cases are in line with a previous study of 120 patients demonstrating no difference in outcome between cases with *GATA2::MECOM* as compared to *MECOM*-r with other partners [12, 13]. Regarding *KMT2A*-r AML, the prognostic impact in ELN guidelines [6, 7] is stratified according to the fusion partner with *KMT2A::MLLT3* indicating intermediate risk and other *KMT2A*-r indicating adverse risk. However, we could not validate this discrimination in our cohort. This could be due to the small sample size of *KMT2A*-r cases (*n* = 45). Notably, in line with our results, another study analyzing 172 *KMT2A*-r AML showed that *KMT2A*-r was associated with adverse outcomes regardless of translocation subtype [14]. Together, our results suggest that AML risk stratification guidelines could assign all *MECOM*-r or *KMT2A*-r cases within the same category.

Another major difference is the definition of AML-MR. In contrast to WHO 2017, WHO 2022 includes defining mutations as entity criteria for AML-MR and does not consider morphologic criteria anymore (same as in the ICC). In line with WHO 2022, ICC also incorporates MR gene mutations (extended to include *RUNX1* mutations). In our cohort 79% (38/48) of previous AML-*RUNX1* harbored either *ASXL1* or splicing mutations in line with previous reports showing the association of *RUNX1* mutations with other genetic features [4, 15]. The AML-MR definition is also heavily affected by the exclusion of *TP53* mutated patients according to ICC. The different MR entity criteria and the introduction of the new ICC entity AML-*TP53* affects not only the composition but also the outcome of AML-MR. The newly defined AML-MR entities potentially impact patient care as CPX-351 (Vyxeos®) was approved amongst others for the treatment of adults with newly diagnosed AML-MRC as defined by WHO 2017 [16–18]. Interestingly, a retrospective study demonstrated that AML patients with *TP53* mutations had poorer responses to CPX-351 [19]. However, whether this drug can also be used for patients diagnosed as AML-MR defined by WHO 2022 and/or ICC is currently not defined, and clarification is needed to ensure usage of this drug in daily practice.

In line with our data, several studies reported a worse prognosis of *TP53* mutated patients [20–23]. However, prognosis alone should not drive disease classification which should rather be based on unique biological characteristics. On the one hand, a uniform prognosis or therapy response might represent a surrogate for a biological group, and the despair prognosis of many of these cases suggests that these patients could theoretically benefit from specific drug development for *TP53* dysregulated AML in the future. On the other hand, *TP53* dysregulation rather represents a common endpoint in different branches of leukemogenesis, and *TP53* mutations should be taken within a broader context of allelic status, mutation number, mutation location within the *TP53* gene, TP53 protein expression, and other concurrent defined genetic alterations [24]. We observed a trend towards worse OS of AML patients with biallelic compared to monoallelic *TP53* mutations. While the number of patients in this cohort is too small for further subgroup analysis, differential effects of the allelic status have been found in larger cohorts [23, 25]. WHO authors did not deem that studies published to date were sufficient for including ‘*TP53* mutated AML’ as a distinct entity as this would result in masking not just AML-MR but other types of AML that may harbor mutant *TP53*. Further studies are needed to definitely answer the open questions including whether all or a subtype of *TP53* altered AML cases should be combined to a new (provisional) entity.

Lastly, one main difference between WHO 2022 and ICC with respect to diagnosing myeloid malignancies is the recognition of the MDS/AML overlap category as a novel disease entity in ICC but not in WHO 2022, affecting 9% of patients within our cohort. As described by Zeidan et al. introducing this novel MDS/AML entity may enable more flexibility for trial enrollment (to either MDS or AML trials) and clinical practice, and may also better reflect the natural history of high-risk MDS [26]. In contrast, the WHO decided not to introduce this overlap category claiming that this may lead to the risk of overtreatment in some patients [2]. However, in individual settings MDS-IB2 may be considered as AML-equivalent for therapy management further arguing for individualized trial enrollment decisions. This seems a reasonable approach as additional validation studies are urgently needed to justify the existence of this new ICC entity. Large data on survival and risk stratification in MDS/AML cohorts as now defined by ICC and ELN are lacking and question at least for now a general eligibility of these patients for AML-based trial designs. Despite this, the different approaches to lower (ICC) or omit (WHO 2022) blast cell cutoffs for the vast majority of genetically defined AML entities affected only a minority of former MDS patients in our cohort that have now been upstaged to AML.

In parallel to the changes in disease classification, adjustments have also been made for risk classification in AML as the new ELN recommendations follow criteria proposed by ICC. This has the potential to substantially complicate diagnosis and treatment of AML patients when applying different in- and exclusion criteria for diagnosis according to WHO 2022 and risk stratification according to ELN 2022. When we compared ELN 2017 and ELN 2022, considerable difference in outcome was only observed for patients falling into the favorable risk group. The later was also shown by Lachowiez et al. analyzing patients of the multi-center Beat AML cohort [27]. However, in contrast to them, we did not observe a better stratification of intermediate or adverse-risk AML patients using ELN 2022 compared to ELN 2017. This may be partly explained as our cohort has not been stratified according to treatment modalities. In addition, the median age was higher in our cohort (68 vs. 62 years) and we observed more patients re-classified from intermediate to adverse risk based on MR-associated gene mutations [27]. Our data are in line with Jentzsch et al. showing that the risk stratification based on the ELN 2022 classification did not significantly improve outcome prognostication compared to the ELN 2017 classification in AML patients undergoing stem cell transplantation [28]. Regarding the overall fit of the model, none of the studies observed a substantial improvement of the c-index by the ELN 2022 compared to ELN 2017 [27, 28].

Our cohort is derived from a single diagnostic laboratory with multiple referring centers. Strengths of this study design include a homogeneous diagnostic workup according to gold standard methods and a powerful genetic analysis with WGS and WTS. Limitations of our study include the heterogeneity in treatment and follow-up assessment in different referral centers. The inclusion of cases diagnosed as early as 2005 provides a long follow-up but reduces the fraction of patients treated with novel therapies. Referral patterns and an enrichment of rare genetic subtypes in our WGS/WTS cohort potentially lead to an overrepresentation of de novo AML in our study compared to other cohorts, and cases diagnosed as therapy-related myeloid neoplasm according to WHO 2017 have not been investigated here.

Overall, we observed a large degree of agreement between the WHO 2022 and the ICC classification with 86% (643/750) AML cases being assigned to corresponding subgroups. The remaining differences are either due to biological aspects/subgroups included in one classification but not the other or by small differences in the inclusion criteria of corresponding subgroups. The first affects a relevant number of patients and should be clarified based on available data to reach a broad consensus. Here, the WHO provides an ideal format to incorporate additional (provisional) entities given that sufficient data were provided. The later affects a relatively small number of patients and should not hamper a common classification. The parallel usage of two different classifications for AML confuses the diagnostic language for physicians, patients and legal authorities. Here it has to be noted that a unified global standard for cancer classification, in which the WHO 2022 is included, is of utmost value for the field. A commonly accepted classification is essential for comparability of diagnostic data in- and outside of clinical studies. We should therefore make all efforts to avoid a Babylonian confusion of languages in the diagnoses of AML and argue for a common diagnostic classification as a foundation to further build upon the tower of knowledge in hematology.

## Supplementary information

Supplemental Material

## Data Availability

The datasets generated during and/or analyzed during the current study are available from the corresponding author on reasonable request.
