# Supplementary material for: AML classification in the year 2023: How to avoid a Babylonian confusion of languages
Source: Leukemia. 2023 Apr 29;37(7):1413–20. doi: 10.1038/s41375-023-01909-w (PMC10317829; doi:10.1038/s41375-023-01909-w)
Supplement: Supplementary file 1 — Supplemental Material [file 41375_2023_1909_MOESM1_ESM.docx]

**Supplementary Material**

**Supplementary Methods**

#### Patients cohort and samples

Diagnoses (from peripheral blood and bone marrow) were made based on cytomorphology, cytogenetics and molecular genetics as previously published [1-3]. Patients were already included in previous studies analyzing *SF3B1* mutations [4, 5].

#### Whole genome and whole transcriptome sequencing (WGS, WTS)

WGS and WTS analysis was performed for all patients. For WGS, total genomic DNA was extracted from lysed cell pellet of bone marrow or peripheral blood using the MagNA Pure 96 with DNA and Viral Nucleic Acid Large Volume Kit and Cellular RNA Large Volume Kit (Roche, Basel, Switzerland). Library preparation and sequencing as well as calling and filtering of single nucleotide variants, structural variants and somatic copy number variations (CNVs) were performed as previously described [6, 7]. Copy neutral loss of heterozygosity (CN-LOH) was assessed using HadoopCNV [8]. WTS was performed as described therein [9]. Fusion calling was performed with Manta (v0.29.0) [10], Arriba (v1.2.0) [11] and STAR-Fusion (v1.9.0) [12].

#### Mutational analysis

Mutational data was retrieved from WGS data or during routine work-up. Structural variants/ fusions were analyzed by combining routine cytogenetics (encompassing chromosome banding analyses and FISH) and WTS.

**Supplementary Results**

**Supplementary Tables and Figures**

**Table S1. WHO 2017 entities of the cohort**

| **WHO 2017 Diagnosis** | **Number of samples** |
| --- | --- |
| **AML** | **734** |
| Acute promyelocytic leukemia with *PML::RARA* (APL) | 48 |
| AML with *RUNX1::RUNX1T1* | 41 |
| AML with *CBFB::MYH11* | 45 |
| AML with *DEK::NUP214* | 15 |
| AML with *KMT2A::MLLT3* | 26 |
| AML with *GATA2::MECOM* | 36 |
| AML with mutated *NPM1* | 162 |
| AML with biallelic mutation of *CEBPA* | 56 |
| AML with mutated *RUNX1* | 48 |
| AML with myelodysplasia-related changes (AML-MRC) | 158 |
| AML with minimal differentiation (FAB M0)* | 10 |
| AML with without maturation (FAB M1)* | 13 |
| AML with maturation (FAB M2)* | 28 |
| Acute myelomonocytic leukemia (FAB M4)* | 33 |
| Acute monoblastic and monocytic leukaemia (FAB M5)* | 14 |
| Pure erythroid leukemia (FAB M6)* | 1 |
| **MDS** | **717** |
| MDS with single lineage dysplasia (MDS-SLD) | 22 |
| MDS with multilineage dysplasia (MDS-MLD) | 96 |
| MDS with single lineage dysplasia with ring sideroblasts (MDS-RS-SLD) | 51 |
| MDS with multilineage dysplasia with ring sideroblasts (MDS-RS-MLD) | 142 |
| MDS with isolated del(5q) (MDS 5q-) | 99 |
| MDS with excess blasts (MDS-EB-1) | 159 |
| MDS with excess blasts (MDS-EB-2) | 148 |
| **Entire cohort** | **1451** |

* summarized as AML, not otherwise specified (AML-NOS)

**Table S2. WHO 2022 entities of the cohort**

| **WHO 2022 Diagnosis** | **Number of samples** |
| --- | --- |
| **AML** | **746** |
| Acute promyelocytic leukemia with *PML::RARA* fusion (APL) | 48 |
| AML with *RUNX1::RUNX1T1* fusion | 41 |
| AML with *CBFB::MYH11* fusion | 45 |
| AML with *DEK::NUP214* fusion | 15 |
| AML with *KMT2A* rearrangement (*KMT2A*-r) | 45 |
| AML with *MECOM* rearrangement (*MECOM*-r) | 69 |
| AML with *NUP98* rearrangement (*NUP98*-r) | 5 |
| AML with *NPM1* mutation | 172 |
| AML with *CEBPA* mutation | 61 |
| AML, myelodysplasia-related (AML-MR) | 208 |
| AML with other defined genetic alterations (AML-ODGA) | 1 |
| AML with minimal differentiation* | 3 |
| AML with without maturation* | 9 |
| AML with maturation* | 10 |
| Acute myelomonocytic leukemia* | 12 |
| Acute monocytic leukaemia* | 1 |
| Acute erythroid leukemia* | 1 |
| **MDS** | **705** |
| MDS with low blasts (MDS-LB) | 119 |
| MDS with low blasts and *SF3B1* mutation (MDS-*SF3B1*) | 180 |
| MDS with low blasts and isolated 5q deletion (MDS-5q) | 98 |
| MDS with biallelic *TP53* inactivation (MDS-bi*TP53*) | 41 |
| MDS with increased blasts-1 (MDS-IB1) | 148 |
| MDS with increased blasts-2 (MDS-IB2) | 119 |
| **Entire cohort** | **1451** |

* summarized as AML defined by differentiation

**Table S3. Cases upstaged from MDS (WHO 2017) to AML (WHO 2022 or ICC)**

| **WHO 2017** | **WHO 2022** | **ICC** | **BM blasts** | **PB blasts** |
| --- | --- | --- | --- | --- |
| MDS-EB-2 | AML with *MECOM*-r ^A^ | MDS/AML with MR gene muts | 18.5 | NA |
| MDS-EB-2 | AML with *MECOM*-r ^A^ | MDS/AML with MR gene muts | 17 | 1 |
| MDS-EB-2 | AML with *MECOM*-r ^A^ | MDS/AML with MR cyto | 11 | 12 |
| MDS-EB-2 | AML with *KMT2A*-r ^A^ | MDS/AML, NOS | 12 | 5 |
| MDS-EB-2 | MDS-IB2 | AML with in-frame bZIP *CEBPA* | 18 ^B^ | 0 |
| MDS-EB-2 | MDS-IB2 | AML with in-frame bZIP *CEBPA* | 15 ^B^ | NA |
| MDS-EB-2 | MDS-IB2 | AML with in-frame bZIP *CEBPA* | 14.5 ^B^ | NA |
| MDS-EB-2 | MDS-IB2 | AML with in-frame bZIP *CEBPA* | 10 ^B^ | NA |
| MDS-EB-2 | AML with *NPM1** | AML with *NPM1** | 16 | NA |
| MDS-EB-2 | AML with *NPM1** | AML with *NPM1** | 15 | 1 |
| MDS-EB-2 | AML with *NPM1** | AML with *NPM1** | 12 | NA |
| MDS-EB-2 | AML with *NPM1** | AML with *NPM1** | 11 | 1 |
| MDS-EB-1 | AML with *NPM1* | MDS-EB | 9 ^C^ | 4 |
| MDS-EB-1 | AML with *MECOM*-r | MDS-EB | 5 ^C^ | NA |
| MDS-RS-MLD | AML with *MECOM*-r | MDS, NOS | 4.5 ^C^ | NA |
| MDS-MLD | AML with *NPM1* | MDS, NOS | 2 ^C^ | NA |

BM: bone marrow; PB: peripheral blood; NA: not available; *consistent between WHO 2022 and ICC; ^A^ specific rearrangement not listed in ICC; ^B^ 20% blast cells cutoff according to WHO 2022 not reached; ^C^ 10% blast cells cutoff according to ICC not reached.

**Table S4. ICC entities of the cohort**

| **ICC Diagnosis** | **Number of samples** |
| --- | --- |
| **AML** | **742** |
| Acute promyelocytic leukemia with *PML::RARA* fusion (APL) | 48 |
| AML with *RUNX1::RUNX1T1* fusion | 41 |
| AML with *CBFB::MYH11* fusion | 45 |
| AML with *DEK::NUP214* fusion | 15 |
| AML with *KMT2A::MLLT3* | 26 |
| AML with other *KMT2A* rearrangements (*KMT2A*-r) | 14 |
| AML with *GATA2::MECOM* | 36 |
| AML with other *MECOM* rearrangements (*MECOM*-r) | 21 |
| AML with mutated *NPM1* | 170 |
| AML with in-frame bZIP *CEBPA* mutations | 47 |
| AML with mutated *TP53* (AML-*TP53*) | 52 |
| AML with MR gene mutations | 174 |
| AML with MR cytogenetic abnormalities | 19 |
| AML, not otherwise specified (AML, NOS) | 34 |
| **MDS/AML** | **137** |
| MDS/AML, not otherwise specified (MDS/AML, NOS) | 13 |
| MDS/AML with MR gene mutations (MDS/AML-mut) | 99 |
| MDS/AML with MR cytogenetic abnormalities (MDS/AML-cyto) | 6 |
| MDS/AML with mutated *TP53* (MDS/AML-*TP53*) | 19 |
| **MDS** | **572** |
| MDS, not otherwise specified (MDS, NOS) | 149 |
| MDS with mutated *SF3B1* (MDS-*SF3B1*) | 153 |
| MDS with del(5q) (MDS-del(5q)) | 98 |
| MDS with mutated *TP53* (MDS-*TP53*) | 22 |
| MDS with excess blasts (MDS-EB) | 150 |
| **Entire cohort** | **1451** |

MR: myelodysplasia-related

**Table S5. Characteristics of AML-*TP53* according to ICC**

| **Characteristics** | **Number of samples (n; %)** |
| --- | --- |
| **AML-*TP53* cases** | **52** |
| Complex karyotype | 46; 88% |
| *TP53* monoallelic | 4; 8% |
| *TP53* biallelic | 48; 92% |
| Two mutations | 11 |
| Mutation + deletion | 25 |
| Mutation + CN-LOH | 12 |
| MR-mutations | 10; 19% |
|  |  |
| ***TP53* mutations** | **63** |
| Mutation type |  |
| Missense | 42 |
| Frameshift | 8 |
| Splice site | 7 |
| Inframe deletion | 3 |
| Nonsense | 2 |
| Inframe insertion | 1 |
| *TP53* VAF |  |
| 10-40% | 8 |
| >40% | 55 |
| Median *TP53* VAF (range) | 63 (17-99%) |

CN-LOH: Copy neutral loss of heterozygosity; VAF: Variant allelic frequency

**Table S6. Changes from ELN 2017 to ELN 2022**

| **Change** | **Number of samples (n)** | **Reason** |
| --- | --- | --- |
| **Downstaging** |  |  |
| Adverse 🡪 intermediate | 2 | *FLT3*-ITDhigh + *NPM1*wt |
| **Upstaging** |  |  |
| Favorable 🡪 intermediate | 14 | *FLT3*-ITDlow + *NPM1*mut |
|  | 6 | Biallelic *CEBPA* without inframe bZIP |
| Favorable 🡪 adverse | 7 | Biallelic *CEBPA* without inframe bZIP + adverse gene mutations |
|  |  |  |
| Intermediate 🡪 adverse | 38 | Adverse gene mutations |
|  | 14 | Adverse chromosomal rearrangements |

**
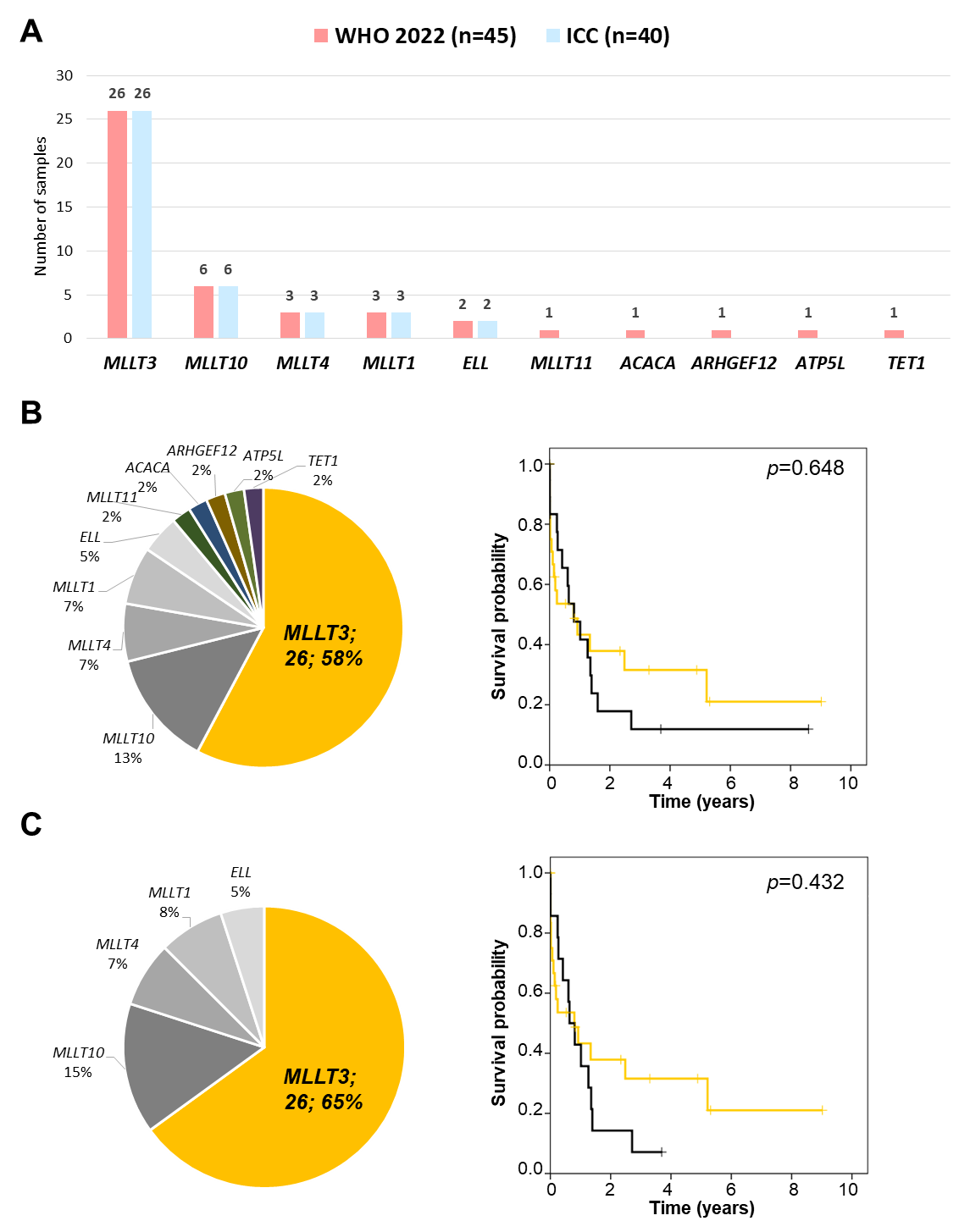
**

**Supplementary Figure S1: Analysis of *KMT2A*-rearranged cases. (A)** Partner genes of *KMT2A*-rearranged cases according to WHO 2022 and ICC. **(B)** Distribution and frequency of *KMT2A*-rearranged cases according to WHO 2022 (n=45) as well as OS of *KMT2A::MLLT3* (yellow; n=26; median OS: 0.8 years) compared to other *KMT2A*-rearranged cases (black: n=19; median OS: 0.8 years). **(C)** Distribution and frequency of *KMT2A*-rearranged cases according to ICC (n=40) as well as OS of *KMT2A::MLLT3* (yellow; n=26; median OS: 0.8 years) compared to other *KMT2A*-rearranged cases (black: n=14; median OS: 0.6 years).

**
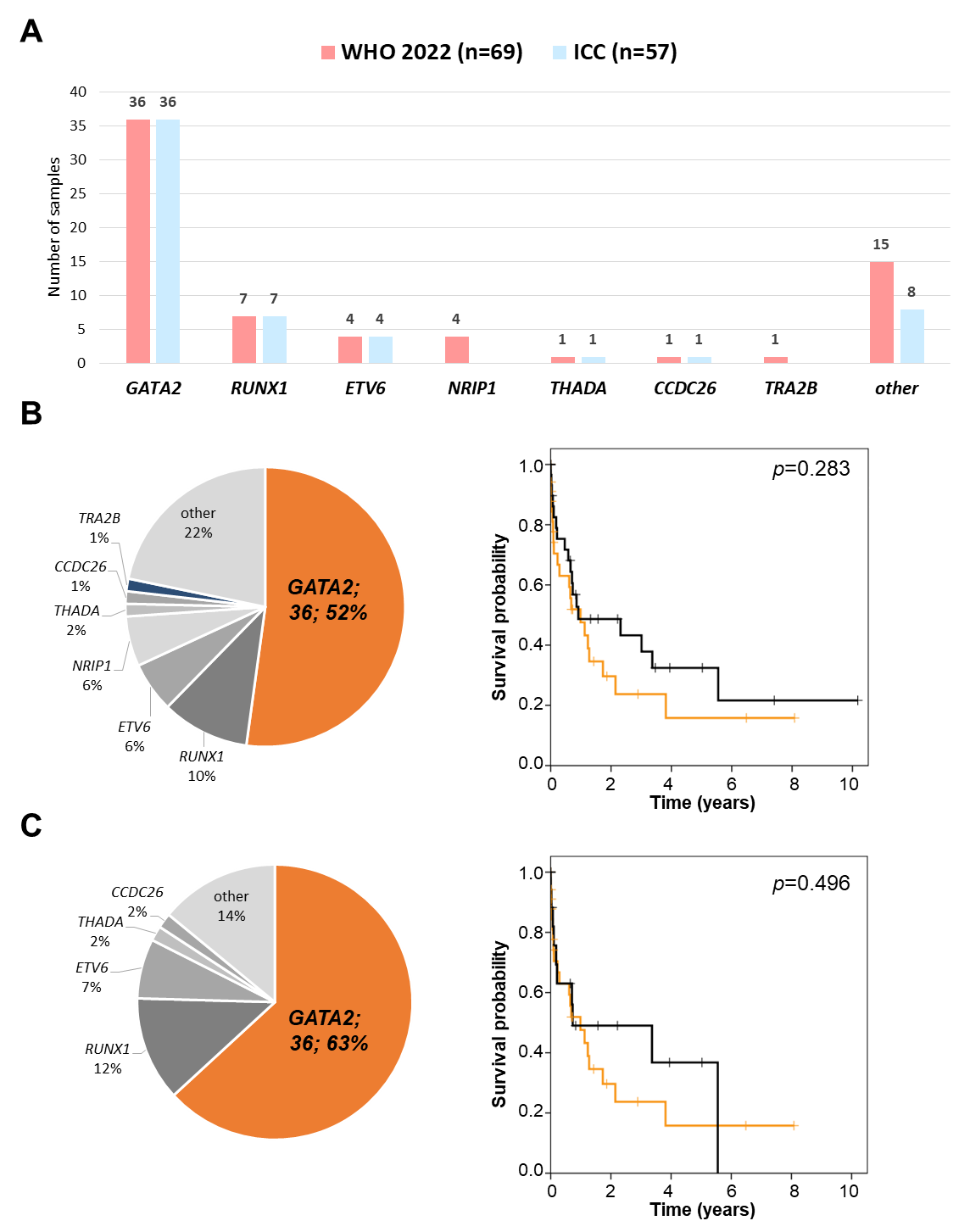
**

**Supplementary Figure S2: Analysis of *MECOM*-rearranged cases. (A)** Partner genes of *MECOM*-rearranged cases according to WHO 2022 and ICC. **(B)** Distribution and frequency of *MECOM*-rearranged cases according to WHO 2022 (n=69) as well as OS of *GATA2::MECOM* (orange; n=36; median OS: 1.0 years) compared to other *MECOM*-rearranged cases (black: n=33; median OS: 0.9 years). **(C)** Distribution and frequency of *MECOM*-rearranged cases according to ICC (n=57) as well as OS of *GATA2::MECOM* (orange; n=21; median OS: 1.0 years) compared to other *MECOM*-rearranged cases (black: n=14; median OS: 0.7 years).


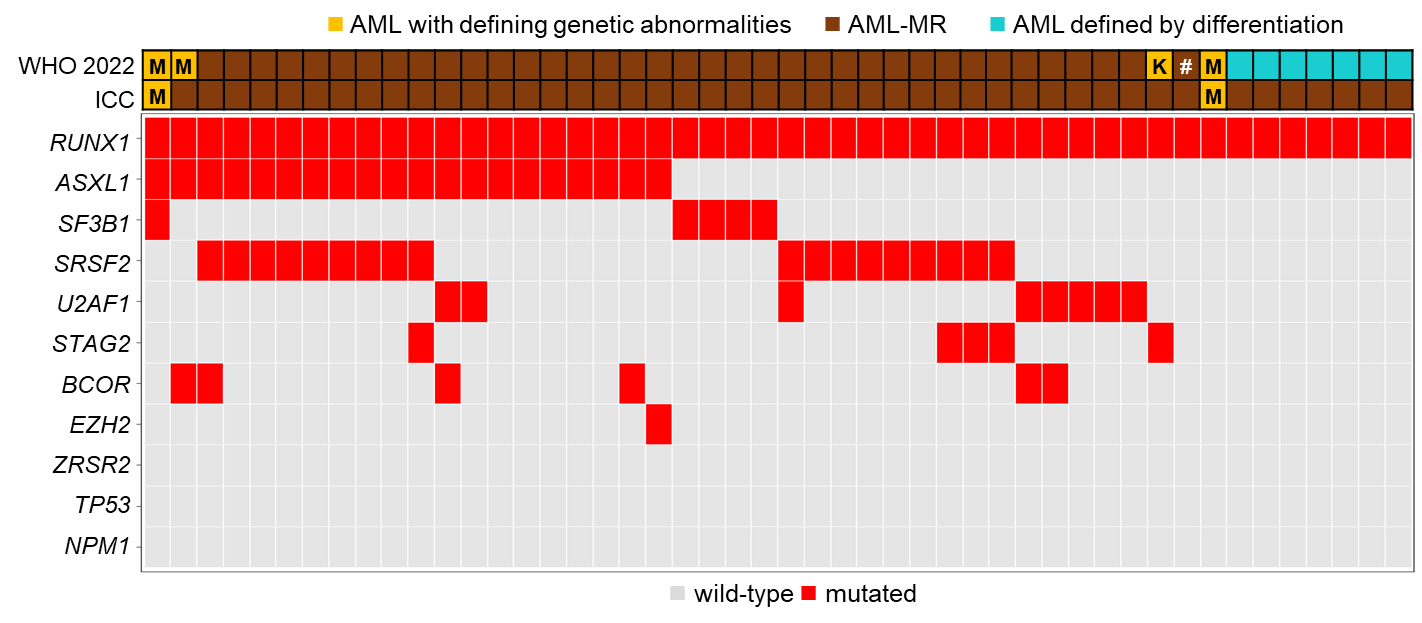


**Supplementary Figure S3: Molecular characterization of patients diagnosed with AML with mutated *RUNX1* according to WHO 2017.** Illustration of all 48 samples, each column represents one patient. AML defining genes (gray: wild-type; red: mutated) as well as the WHO 2022 and ICC entities are given for each patient. MR: myelodysplasia-related; M: *MECOM* rearrangement; K: *KMT2A* rearrangement; #: complex karyotype.


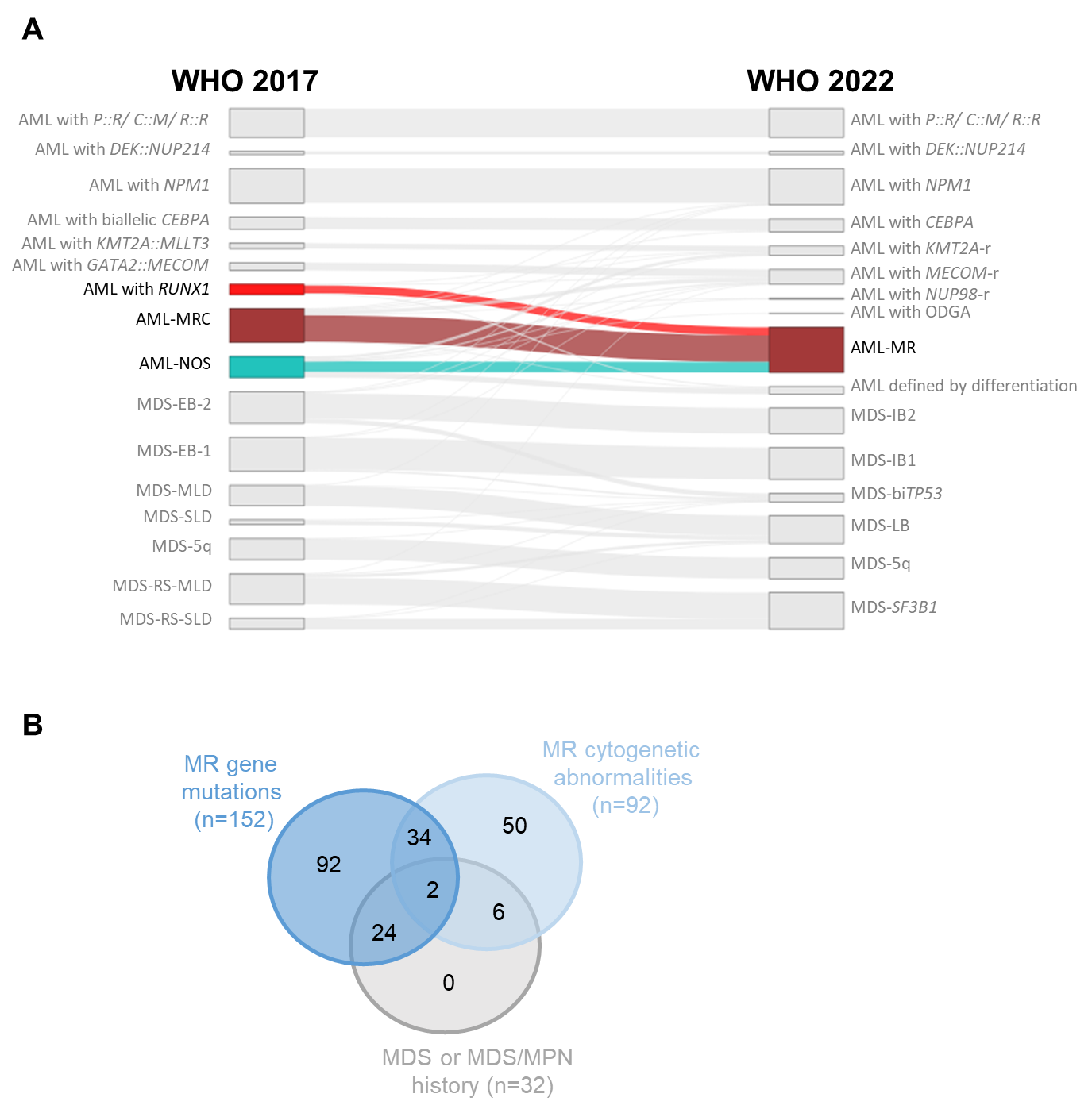


**Supplementary Figure S4: Myelodysplasia-related AML based on WHO 2022. (A)** Composition of AML-MR based on WHO 2022 and corresponding former entities according to WHO 2017. *P::R* = *PML::RARA*; *C::M* = *CBFB::MYH11*; *R::R* = *RUNX1::RUNX1T1*; MR(C): myelodysplasia-related (changes); NOS: not otherwise specified; EB: excess blasts; SLD: single lineage dysplasia; MLD: multilineage dysplasia; 5q: isolated 5q deletion; RS: ring sideroblasts; -r: rearrangement; ODGA: other defined genetic alterations; IB: increased blasts; bi*TP53*: biallelic *TP53* inactivation; LB: low blasts. **(B)** Characteristics/ entity criteria of AML-MR based on WHO 2022 (n=208).


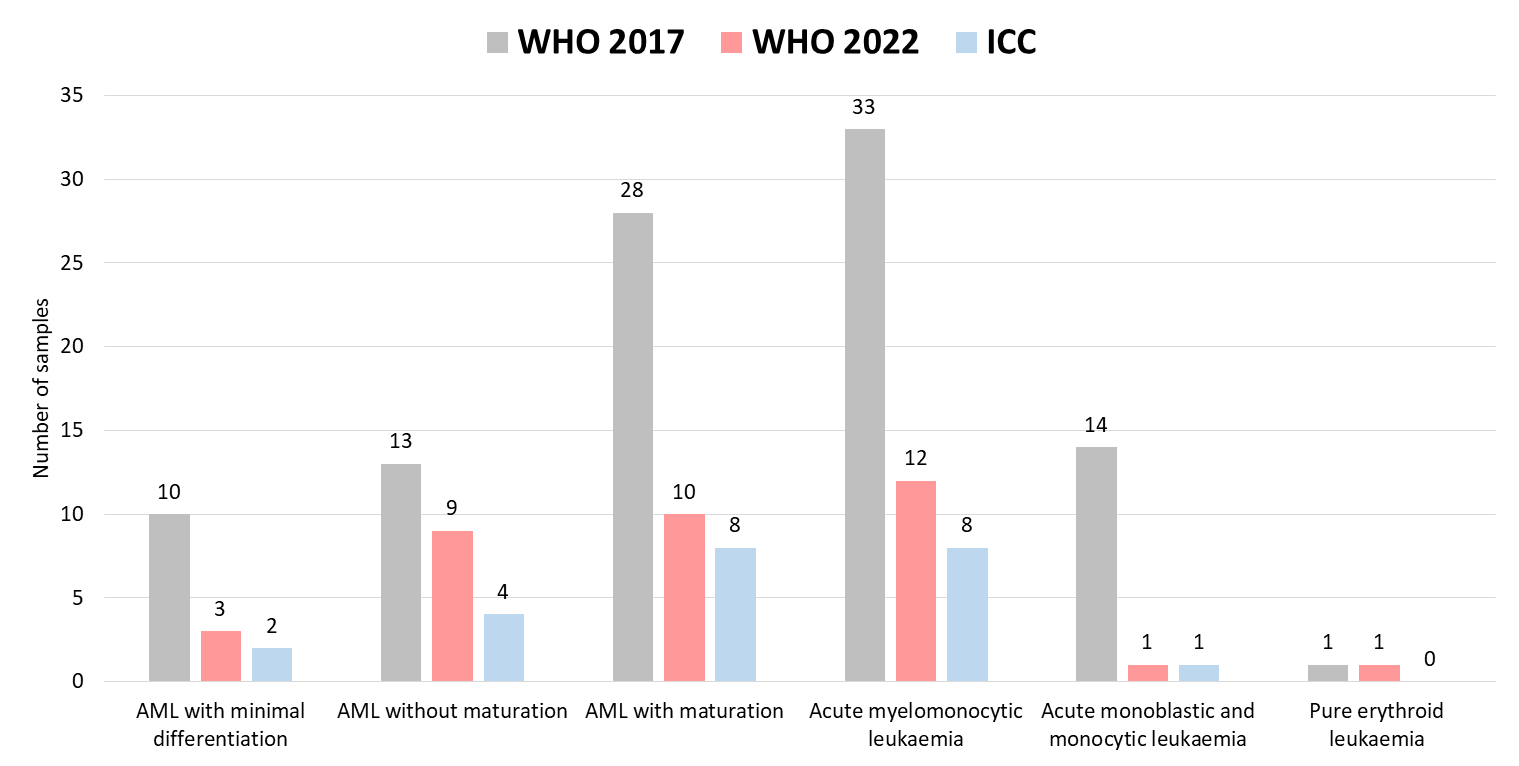


**Supplementary Figure S5: Distribution of morphologically defined AML subgroups according to WHO 2017 (n=99), WHO 2022 (n=36) and ICC (n=34*).** *11/34 were not classified as AML-NOS based on WHO 2017.

**
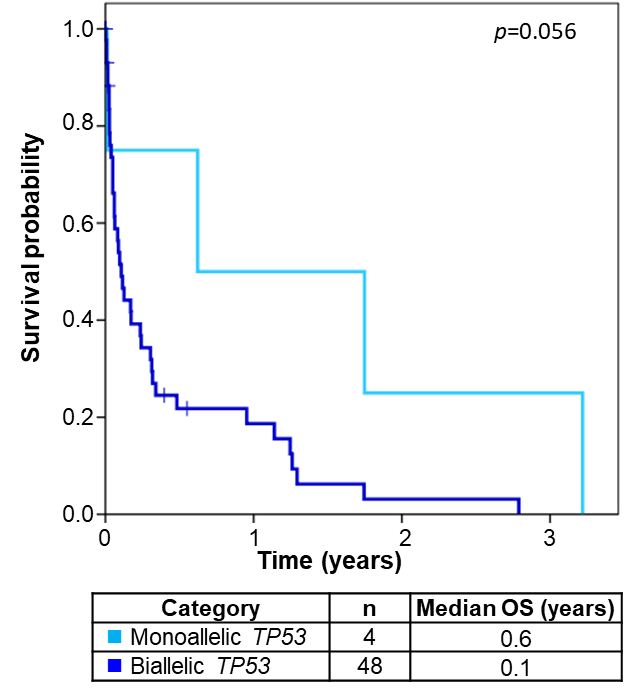
**

**Supplementary Figure S6: OS of AML-*TP53* based on ICC.** OS of AML-*TP53* patients based on ICC (n=52) according to *TP53* mutation status (monoallelic: dark blue, n=48; biallelic: light blue, n=4).

**
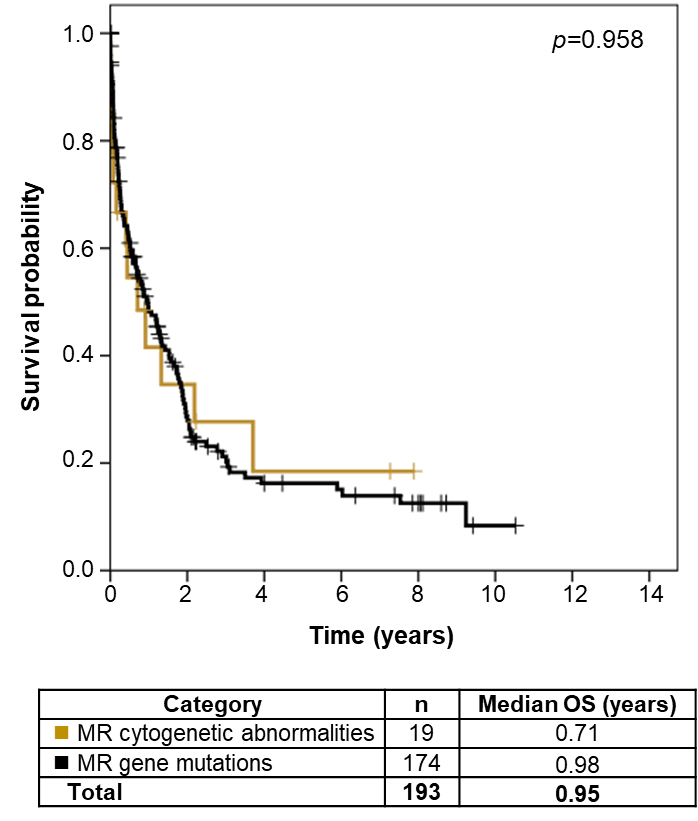
**

**Supplementary Figure S7: OS of subgroups of myelodysplasia-related AML based on ICC.** OS of AML-MR patients based on ICC (n=193) according to AML with MR gene mutations (black: n=174) or AML with MR cytogenetic abnormalities (brown: n=19).


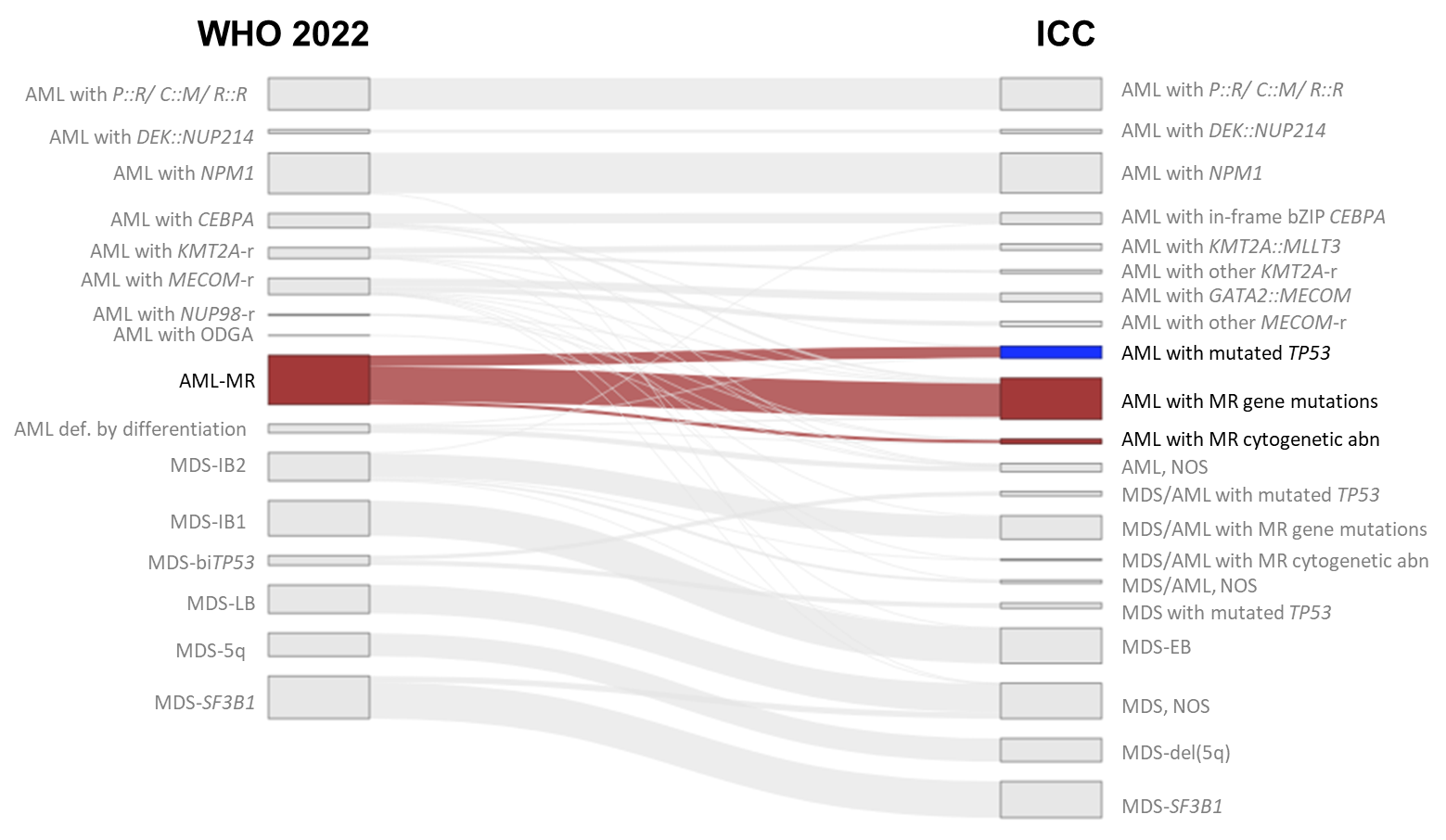


**Supplementary Figure S8: Composition of AML-MR based on WHO 2022 and corresponding entities according to ICC.** *P::R* = *PML::RARA*; *C::M* = *CBFB::MYH11*; *R::R* = *RUNX1::RUNX1T1*; MR(C): myelodysplasia-related (changes); NOS: not otherwise specified; EB: excess blasts; SLD: single lineage dysplasia; MLD: multilineage dysplasia; 5q/del(5q): isolated 5q deletion; RS: ring sideroblasts; -r: rearrangement; ODGA: other defined genetic alterations; IB: increased blasts; bi*TP53*: biallelic *TP53* inactivation; LB: low blasts; abn: abnormalities.


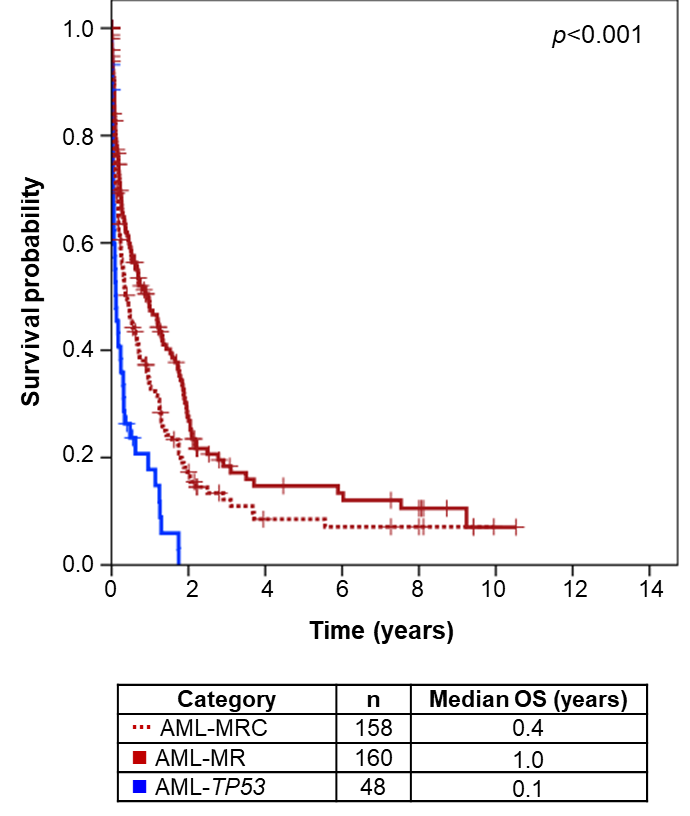


**Supplementary Figure S9: OS of myelodysplasia-related AML.** OS of AML-MR patients based on WHO 2022 (n=208) according to corresponding ICC entities AML-MR (dark red; n=160) or AML-*TP53* (blue; n=48) compared to AML-MRC based on WHO 2017 (dark red, dotted line; n=158).

**
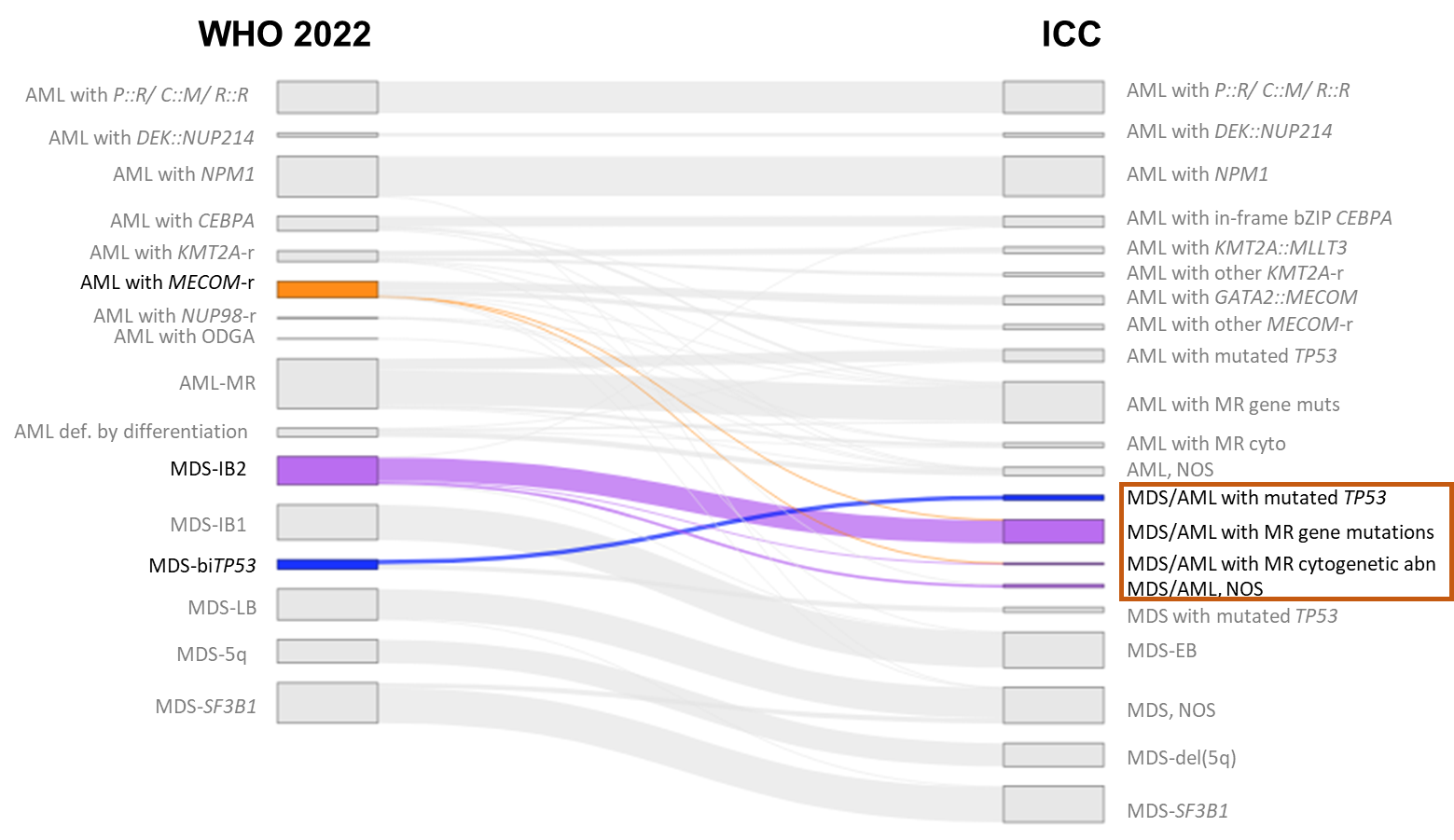
**

**Supplementary Figure S10: Composition of MDS/AML based on ICC and corresponding entities according to WHO 2022.** *P::R* = *PML::RARA*; *C::M* = *CBFB::MYH11*; *R::R* = *RUNX1::RUNX1T1*; MR(C): myelodysplasia-related (changes); NOS: not otherwise specified; EB: excess blasts; SLD: single lineage dysplasia; MLD: multilineage dysplasia; 5q/del(5q): isolated 5q deletion; RS: ring sideroblasts; -r: rearrangement; ODGA: other defined genetic alterations; IB: increased blasts; bi*TP53*: biallelic *TP53* inactivation; LB: low blasts; abn: abnormalities.

**
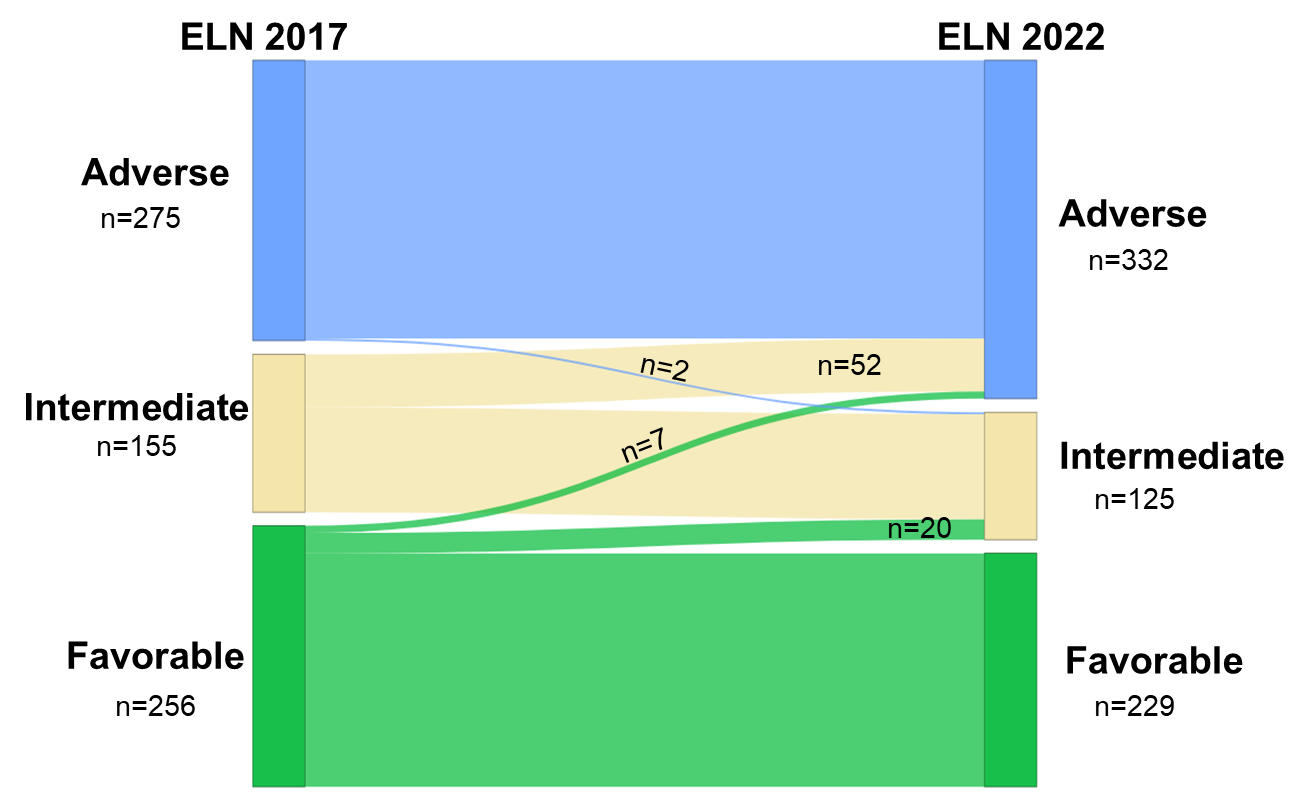
**

**Supplementary Figure S11: Changes in AML risk classification according to different ELN guidelines.** ELN risk classifications of AML patients based on WHO 2017 (n=686; excluding AML with *PML::RARA*) are depicted.

## References

1. Schoch C, Schnittger S, Bursch S, Gerstner D, Hochhaus A, Berger U, et al. Comparison of chromosome banding analysis, interphase- and hypermetaphase-FISH, qualitative and quantitative PCR for diagnosis and for follow-up in chronic myeloid leukemia: a study on 350 cases. Leukemia. 2002;16(1):53-9.

2. Haferlach T, Kern W, Schoch C, Hiddemann W, Sauerland MC. Morphologic dysplasia in acute myeloid leukemia: importance of granulocytic dysplasia. J Clin Oncol. 2003;21(15):3004-5.

3. Kern W, Voskova D, Schoch C, Hiddemann W, Schnittger S, Haferlach T. Determination of relapse risk based on assessment of minimal residual disease during complete remission by multiparameter flow cytometry in unselected patients with acute myeloid leukemia. Blood. 2004;104(10):3078-85.

4. Huber S, Haferlach T, Meggendorfer M, Hutter S, Hoermann G, Baer C, et al. SF3B1 mutated MDS: Blast count, genetic co-abnormalities and their impact on classification and prognosis. Leukemia. 2022. doi: 10.1038/s41375-022-01728-5

5. Huber S, Haferlach T, Meggendorfer M, Hutter S, Hoermann G, Baer C, et al. SF3B1 mutations in AML are strongly associated with MECOM rearrangements and may be indicative of an MDS pre-phase. Leukemia. 2022. doi: 10.1038/s41375-022-01734-7

6. Höllein A, Twardziok SO, Walter W, Hutter S, Baer C, Hernandez-Sanchez JM, et al. The combination of WGS and RNA-Seq is superior to conventional diagnostic tests in multiple myeloma: Ready for prime time? Cancer Genet. 2020;242:15-24.

7. Stengel A, Baer C, Walter W, Meggendorfer M, Kern W, Haferlach T, et al. Mutational patterns and their correlation to CHIP-related mutations and age in hematological malignancies. Blood Adv. 2021;5(21):4426-34.

8. Yang H, Chen G, Lima L, Fang H, Jimenez L, Li M, et al. HadoopCNV: A dynamic programming imputation algorithm to detect copy number variants from sequencing data. bioRxiv. 2017:124339.

9. Stengel A, Shahswar R, Haferlach T, Walter W, Hutter S, Meggendorfer M, et al. Whole transcriptome sequencing detects a large number of novel fusion transcripts in patients with AML and MDS. Blood Adv. 2020;4(21):5393-401.

10. Chen X, Schulz-Trieglaff O, Shaw R, Barnes B, Schlesinger F, Källberg M, et al. Manta: rapid detection of structural variants and indels for germline and cancer sequencing applications. Bioinformatics. 2016;32(8):1220-2.

11. Uhrig S, Ellermann J, Walther T, Burkhardt P, Fröhlich M, Hutter B, et al. Accurate and efficient detection of gene fusions from RNA sequencing data. Genome Research. 2021;31(3):448-60.

12. Haas BJ, Dobin A, Li B, Stransky N, Pochet N, Regev A. Accuracy assessment of fusion transcript detection via read-mapping and de novo fusion transcript assembly-based methods. Genome Biology. 2019;20(1):213.
